# Anesthetic Management of Asymptomatic Innominate Artery Compression Syndrome in an Elderly Patient Using Near-Infrared Spectroscopy and Bilateral Radial Arterial Pressure Monitoring

**DOI:** 10.7759/cureus.108867

**Published:** 2026-05-14

**Authors:** Misato Kanematsu, Tatsuya Tsuji, Rei Tsuji

**Affiliations:** 1 Anesthesiology, Okazaki City Hospital, Okazaki, JPN

**Keywords:** airway management, high-flow humidified nasal cannula therapy, innominate artery compression syndrome, near-infrared spectroscopy, tracheal intubation

## Abstract

Innominate artery compression syndrome (IACS) is rare in adults and poses anesthetic challenges, as airway manipulation may exacerbate tracheal compression or compromise cerebral perfusion. Peri-intubation cerebral oxygenation in this context has rarely been reported.

We describe the case of an 82-year-old woman with incidentally detected, asymptomatic IACS and moderate tracheal stenosis who underwent robot-assisted rectal surgery under general anesthesia. During fiberoptic-guided tracheal intubation, bilateral radial arterial pressures and near-infrared spectroscopy (NIRS) were continuously monitored to assess vascular and cerebral perfusion. No decline in regional cerebral oxygen saturation (rSO_2_) or increase in bilateral arterial pressure gradient was observed, and ventilation remained stable, including during steep Trendelenburg positioning.

Careful anesthetic planning, with continuous monitoring of cerebral oxygenation and bilateral arterial pressures, may facilitate safe perioperative management in adults with this syndrome.

## Introduction

Vascular rings and vascular-related aerodigestive compression syndromes comprise a spectrum of congenital arch anomalies that compress the trachea, esophagus, or both. True, or complete, vascular rings include double aortic arch and right aortic arch with an aberrant left subclavian artery and left ligamentum arteriosum, with a reported prevalence of approximately 1-1.3 per 10,000 live births [1]. These lesions typically present in infancy with wheezing and recurrent respiratory infections [2]. In contrast, adult presentations are extremely rare and may be associated with congenital vascular anomalies, arterial aneurysms, or arterial tortuosity [3]. Innominate artery compression syndrome (IACS) is a rare condition, in which an anomalous or dilated innominate artery compresses the trachea. Adult management remains poorly defined, and failure to recognize IACS preoperatively may create substantial airway risk after anesthesia induction [4-7].

Previous literature has largely emphasized airway management strategies in adults with IACS [3], while the potential effects of airway manipulation on cerebral perfusion remain insufficiently characterized. Although reports of overt cerebral ischemia directly attributable to IACS are limited, the close anatomical relationship between the trachea and innominate artery suggests that airway maneuvers could theoretically compress this vessel. Because the innominate artery supplies the right common carotid and right subclavian arteries, such compression or altered hemodynamics could compromise cerebral blood flow. Surgical cross-clamping of the innominate artery carries a risk of cerebral ischemia when collateral circulation is inadequate; therefore, continuous monitoring of regional cerebral oxygen saturation (rSO₂) may help identify evolving hypoperfusion and guide safer perioperative management [8]. The reflected light attenuation represents information regarding rSO₂ and the balance between oxygen delivery and oxygen consumption, making near-infrared spectroscopy (NIRS) a very sensitive technology to changes in cerebral oxygenation [9]. Even non-surgical interventions, such as transesophageal echocardiography (TEE) probe insertion, can mechanically compress anomalous arch vessels and induce cerebral hypoperfusion [10]. Changes in head and neck position for optimal laryngoscopy, including the sniffing position, may also independently influence cerebral blood flow [11].

On this basis, we hypothesized that mechanical stimuli and structural displacement associated with standard airway management - including the sniffing position, endotracheal tube advancement, and cuff inflation - could exert additive compressive forces on the innominate artery and compromise cerebral perfusion. We describe the anesthetic management of an elderly patient with asymptomatic IACS incidentally detected on preoperative CT, with particular emphasis on airway management and perioperative monitoring of cerebral oxygenation using NIRS and bilateral radial arterial pressure measurement. Written informed consent was obtained from the patient for publication of this case report and accompanying images.

## Case presentation

An 82-year-old woman (weight: 67 kg; height: 145 cm) had a medical history of moderate aortic stenosis, liver cirrhosis, and esophageal varices. Her cirrhosis was classified as Child-Pugh class A [12], with a Model for End-Stage Liver Disease score of 3 [13]. Endoscopy revealed extensive esophagogastric varices extending to the upper esophagus, without red color signs suggestive of imminent bleeding. Transthoracic echocardiography confirmed moderate aortic stenosis, showing a peak transvalvular velocity of 3.9 m/s, a mean pressure gradient of 35 mmHg, and an aortic valve area of 0.90 cm². She was scheduled for robot-assisted low anterior resection for rectal cancer.

**Figure 1:**
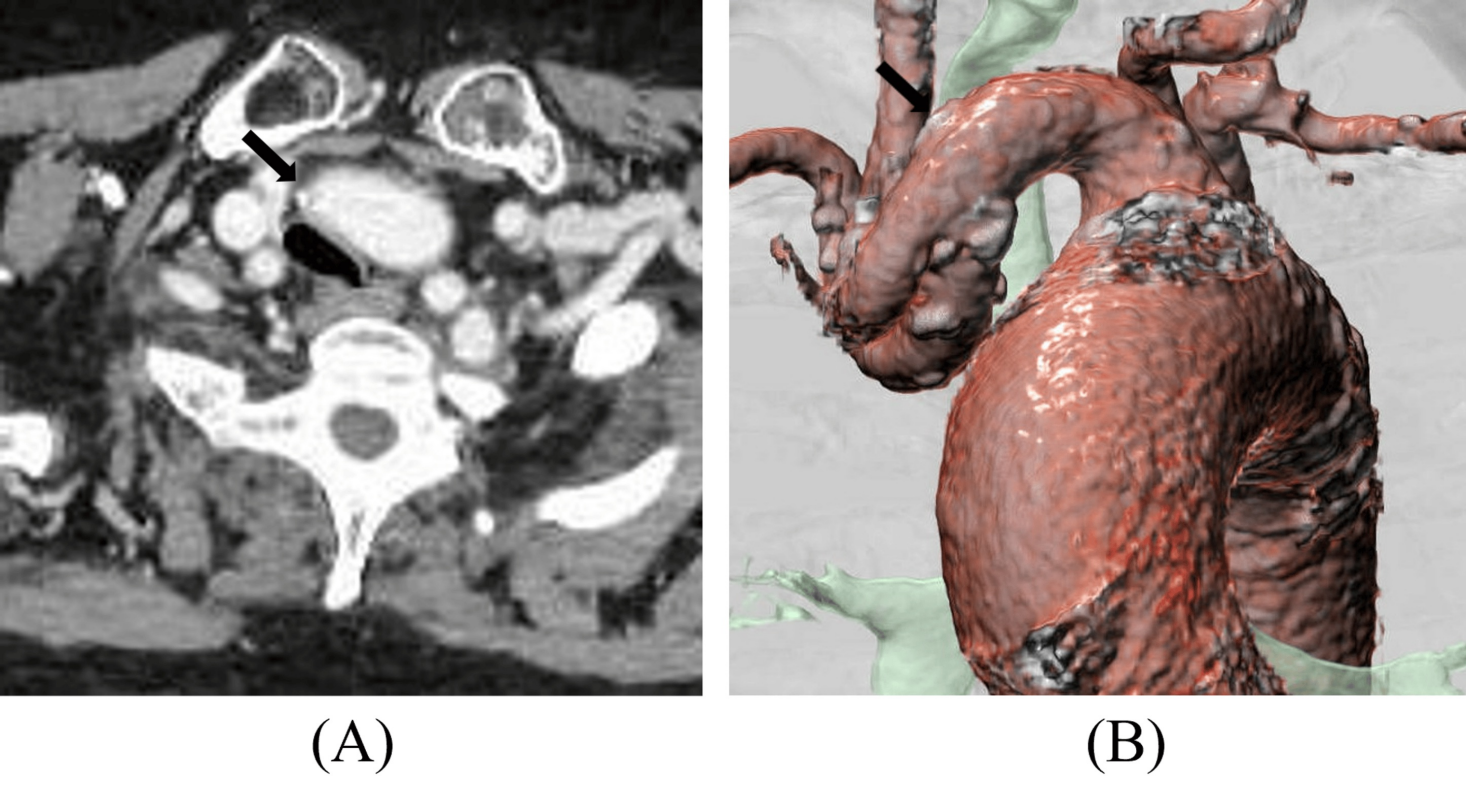
Contrast-enhanced and 3D chest CT showing tracheal compression by the innominate artery (A) Contrast-enhanced CT showing a suspected bovine aortic arch, and compression of the trachea by a tortuous innominate artery; (B) 3D CT reconstruction demonstrating the suspected bovine aortic arch, with the trachea compressed and narrowed by the innominate artery. Arrows indicate the innominate artery.

Preoperative contrast-enhanced chest CT, performed as part of routine staging for rectal cancer, showed a suspected bovine aortic arch with tracheal compression caused by a tortuous innominate artery, consistent with IACS (Figure 1).

The narrowest tracheal diameter was 8 mm, corresponding to 57% stenosis. Pulmonary function testing showed a forced vital capacity (FVC) of 1.53 L (78% predicted), forced expiratory volume in 1 s (FEV₁) of 1.29 L, and an FEV₁/FVC ratio of 80%, consistent with mild restrictive impairment.

A multidisciplinary conference was convened to evaluate the risks of difficult intubation, cerebral hypoperfusion, and hemorrhage secondary to innominate artery injury. Because the patient was asymptomatic, tolerated the supine position, and had only moderate stenosis, prophylactic surgery or standby extracorporeal membrane oxygenation was deemed unnecessarily invasive. Accordingly, an individualized anesthetic plan was developed.

In the operating room, standard monitoring was applied. NIRS (NIRO-200NX, Hamamatsu Photonics K.K., Japan) sensors were placed on the forehead. Bilateral radial arterial lines were established for continuous comparison of right- and left-sided arterial pressures. A decrease in rSO₂ of 20% or more from baseline was defined as clinically significant, and a left-right discrepancy of 15 mmHg or more in bilateral radial arterial pressures was considered significant [14,15]. If either threshold was reached, the contingency plan was immediate tracheal tube withdrawal and conversion to supraglottic airway management. After preoxygenation with high-flow humidified nasal cannula (HFNC) (Steady Air, Atom Medical Co., Japan), anesthesia was induced with remimazolam (6 mg/kg/h), fentanyl 100 µg, and rocuronium 50 mg.

**Figure 2:**
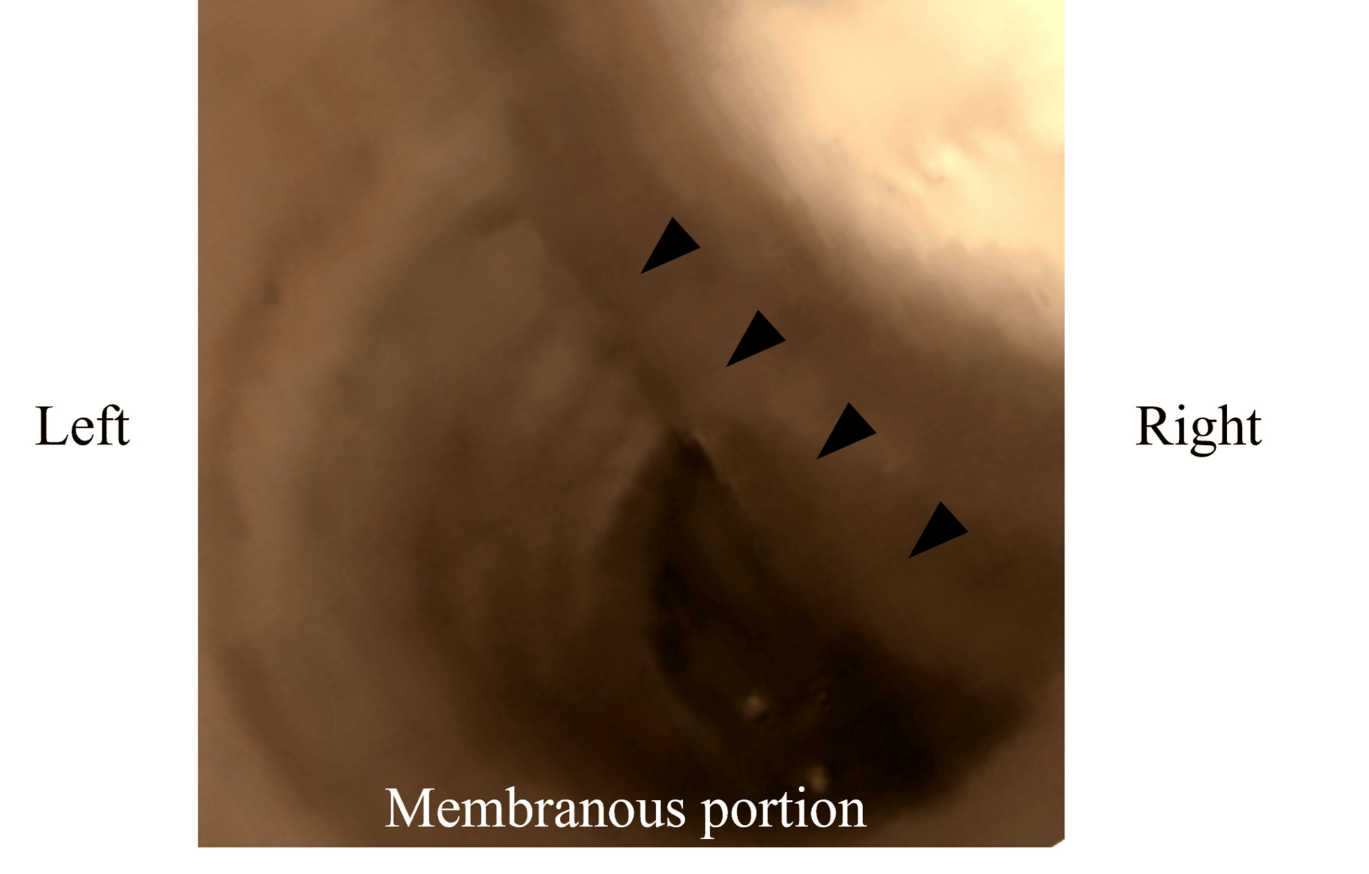
Bronchoscopy showing pulsatile right anterior tracheal wall compression by the innominate artery Arrows indicate the innominate artery.

Following confirmation of mask ventilation, a reinforced tracheal tube (6.0 mm ID; Shiley Lo-Contour, Covidien, USA) was advanced distal to the stenosis under fiberoptic guidance (VATHiN; TOKIBO, Japan). Pulsatile compression of the anterior tracheal wall by the innominate artery was observed (Figure 2).

**Figure 3:**
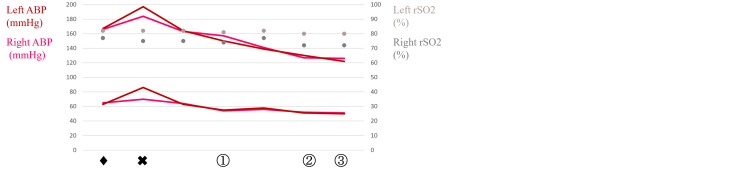
Changes in rSO₂ and bilateral radial arterial pressures during anesthesia induction ♦: Arrival in the operating room; ✖: Application of HFNC and start of anesthesia; ①: Head-up positioning and initiation of anesthetic administration; ②: Fiberoptic-guided intubation with a reinforced tracheal tube; ③: Check the patient's position in the Trendelenburg position ABP: Arterial blood pressure; rSO₂: Regional cerebral oxygen saturation; HFNC: High-flow humidified nasal cannula

Continuous monitoring showed no significant left-right discrepancy in cerebral oxygenation or arterial pressure. Pre-intubation rSO₂ values were 75% (right) and 81% (left) with symmetric radial pressures (171/63 mmHg and 171/62 mmHg). After tube placement and cuff inflation, rSO₂ remained stable (72% right, 81% left) and blood pressures remained symmetric. Ventilation was maintained without hypercapnia. A 20° Trendelenburg tilt did not compromise ventilation, while rSO₂ was maintained and blood pressures remained symmetric. A right internal jugular central venous catheter was then placed. The left-right differences in rSO₂ and bilateral radial arterial pressures remained stable throughout induction (Figure 3).

The operative time was 5 hours 53 minutes, with anesthesia lasting 8 hours 5 minutes. At the end of surgery, the tracheal tube was exchanged for a supraglottic airway device (i-gel; Intersurgical, UK). Bronchoscopic evaluation before extubation confirmed no progression of airway edema or stenosis. Postoperatively, the patient was admitted to the ICU for overnight observation. Cerebral oxygenation monitoring was discontinued at the end of anesthesia, and arterial lines were maintained until ICU discharge. No neurological deficits suggestive of cerebral ischemia were observed. The patient was transferred to a chronic care hospital on postoperative day 20 after an uncomplicated postoperative course.

## Discussion

The potential effects of airway manipulation on cerebral perfusion in adult IACS have not been systematically evaluated. However, available evidence supports a plausible risk of iatrogenic vascular compression. First, changes in head and neck position, such as the sniffing position, can independently reduce rSO₂ [11]. Second, TEE probe insertion has been reported to mechanically compromise anomalous arch vessels and reduce rSO₂ [10]. Third, experience with surgical cross-clamping demonstrates that the innominate artery supplies critical inflow to the right cerebral hemisphere and right upper extremity; therefore, obstruction may cause right-sided ischemia when collateral circulation is inadequate [8]. Collectively, these findings suggest that standard airway management maneuvers - including the sniffing position, tracheal tube advancement, and cuff inflation - could exert additive compressive forces on the innominate artery and precipitate right-sided cerebral ischemia.

On this physiological basis, continuous monitoring of rSO₂ and bilateral radial arterial pressure was considered a clinically useful adjunct to detect evolving cerebral hypoperfusion or innominate artery compromise. As the right radial artery arises from the right subclavian artery, innominate artery compression would be expected to produce an isolated right-sided pressure decrease or a left-right pressure discrepancy. In our patient, both parameters remained stable throughout induction and intubation, suggesting that airway management did not critically compromise the innominate artery, likely because of the moderate stenosis (57%) and adequate collateral circulation.

Although no perfusion impairment occurred, establishing a contingency plan remains essential. If a significant decrease in rSO₂ (>20% from baseline) or a marked blood pressure discrepancy had occurred, the protocol called for immediate withdrawal of the tracheal tube and conversion to a supraglottic airway device. Preoxygenation with high-flow nasal cannula and post-induction assessment in the Trendelenburg position provided additional safety margins against ventilatory failure [11]. Although the patient in our case was asymptomatic, IACS typically presents with various respiratory symptoms-including dyspnea, wheezing, cough, and recurrent respiratory infections-and is therefore frequently misdiagnosed as other respiratory conditions [2]. Therefore, when encountering patients who present with such symptoms, we suggest that anesthesiologists carefully review preoperative CT images. Furthermore, even in the absence of overt symptoms, routine evaluation of available preoperative CT scans may facilitate early detection of underlying anatomical anomalies such as IACS, which can pose rare but significant airway risks.

Several limitations should be acknowledged. First, NIRS provides regional rather than global perfusion data, and rSO₂ is influenced by systemic hemodynamics. Second, given the moderate stenosis, standard airway management may have been sufficient even without intensive monitoring. Finally, as a single case report, neither definitive ischemic thresholds nor the diagnostic accuracy of NIRS for detecting perfusion compromise in IACS can be determined. This case, however, supports the feasibility of using real-time monitoring of rSO₂ and bilateral radial arterial pressure measurement to detect potential cerebral perfusion impairment during airway management. Additional cases are needed to clarify the clinical significance of these monitoring strategies and optimize anesthetic strategies for adult patients with IACS.

## Conclusions

Although asymptomatic IACS is rare in adults, standard airway manipulation may carry a theoretical risk of inducing iatrogenic cerebral ischemia. In the present case, continuous monitoring of rSO_2_ and bilateral radial arterial pressures provided real-time information regarding cerebral perfusion. While further clinical evidence is needed to determine the risk-benefit ratio and necessity of such invasive measures, this case demonstrates the feasibility of using tailored multimodal monitoring to observe cerebral hemodynamics during airway management in patients with this vascular anomaly.
